# Introducing a Method for Calculating the Allocation of Attention in a Cognitive “Two-Armed Bandit” Procedure: Probability Matching Gives Way to Maximizing

**DOI:** 10.3389/fpsyg.2016.00223

**Published:** 2016-03-08

**Authors:** Gene M. Heyman, Katherine A. Grisanzio, Victor Liang

**Affiliations:** ^1^Department of Psychology, Boston College, Chestnut HillMA, USA; ^2^Department of Biochemistry, Boston College, Chestnut HillMA, USA

**Keywords:** attention allocation, optimizing, reward, learned predictiveness, mathematical model

## Abstract

We tested whether principles that describe the allocation of overt behavior, as in choice experiments, also describe the allocation of cognition, as in attention experiments. Our procedure is a cognitive version of the “two-armed bandit choice procedure.” The two-armed bandit procedure has been of interest to psychologistsand economists because it tends to support patterns of responding that are suboptimal. Each of two alternatives provides rewards according to fixed probabilities. The optimal solution is to choose the alternative with the higher probability of reward on each trial. However, subjects often allocate responses so that the probability of a response approximates its probability of reward. Although it is this result which has attracted most interest, probability matching is not always observed. As a function of monetary incentives, practice, and individual differences, subjects tend to deviate from probability matching toward exclusive preference, as predicted by maximizing. In our version of the two-armed bandit procedure, the monitor briefly displayed two, small adjacent stimuli that predicted correct responses according to fixed probabilities, as in a two-armed bandit procedure. We show that in this setting, a simple linear equation describes the relationship between attention and correct responses, and that the equation’s solution is the allocation of attention between the two stimuli. The calculations showed that attention allocation varied as a function of the degree to which the stimuli predicted correct responses. Linear regression revealed a strong correlation (*r* = 0.99) between the predictiveness of a stimulus and the probability of attending to it. Nevertheless there were deviations from probability matching, and although small, they were systematic and statistically significant. As in choice studies, attention allocation deviated toward maximizing as a function of practice, feedback, and incentives. Our approach also predicts the frequency of correct guesses and the relationship between attention allocation and response latencies. The results were consistent with these two predictions, the assumptions of the equations used to calculate attention allocation, and recent studies which show that predictiveness and reward are important determinants of attention.

## Introduction

We are interested in whether there are general quantitative principles that apply to both the allocation of overt behavior and the allocation of cognition. For example, in analogous cognitive and behavioral experiments, do individuals maximize correct responses, and, if so, are the mediating processes the same or similar? Such questions are challenging, because they presuppose that we can measure the allocation of cognition with the same precision as we measure the allocation of choices, say on a scale that varies from 0.0 to 1.0. Although there are many approaches to the measurement of attention, including a host of quantitative models (e.g., see Bundesen, 1996 and Logan, 2004 for reviews), we found none that provided a continuous numerical scale for the division of attention. This is important; it is often the case that qualitatively different principles predict similar yet not identical quantitative outcomes. For example, molar maximizing, as in economic texts (e.g., Baumol and Blinder, 1994), local maximizing, as in the matching law (e.g., Herrnstein, 2000), and probability matching (e.g., Estes, 1976) predict similar quantitative results under some conditions yet are different principles, reflecting fundamentally different choice rules (see, Heyman and Luce, 1979; Herrnstein, 2000). Thus, our first task was to develop a method that would provide a continuous measure of the allocation of attention.

In the Section “Materials and Methods”, we describe our approach to this problem. It combines widely used methods in research on attention and choice (Estes, 1976; Corbetta et al., 1990; Le Pelley et al., 2013) with a mathematical model of performance in the procedure. The simplest version of the model is a single variable linear equation, whose solution is our desired result, the allocation of attention. Thus, the new feature of the procedure is the equation for calculating attention allocation. The results support the conclusions that the equation accurately described the relative amount of attention devoted to each of two simultaneously available visual stimuli and that the allocation of attention shifted over the course of two sessions so as to increase the number of correct answers.

The theoretical background for this study includes widely shared ideas regarding adaptive behavior, and the empirical background includes recent experiments on the role of reward and predictiveness in attention. First, the hypothesis that natural phenomena can be understood as solutions to optimizing problems has proven useful throughout the sciences. Second, it is not unreasonable to suppose that there may be general principles that govern both the allocation of attention and the allocation of overt behavior. Third, in recent years several research groups have published studies on how reward influences the allocation of attention (Small et al., 2005; Anderson and Yantis, 2013; Chelazzi et al., 2013; Lee and Shomstein, 2014). For instance, subjects are less likely to report the second of two successive stimuli if the lag between them is about 150–400 ms, a phenomenon referred to as “attentional blink.” However, if the second signal predicted a valued reward, performance at a 200 ms lag was about as good as at an 800 ms lag (Raymond and O’Brien, 2009). In other words, reward markedly reduced the attentional blink effect. Fourth, recent studies show that predictiveness can come to control attention, where predictiveness refers to the correlation between a stimulus and a valued outcome, independent of whether the value is low or high (e.g., Le Pelley et al., 2013). Formal models of the role of predictiveness in attention borrow heavily from the learning literature (e.g., Esber and Haselgrove, 2011), and, on the basis of a recent experiment, Le Pelley et al. (2013) suggest that that predictiveness may capture attention in a rapid, automatic fashion, as do highly salient features of the physical world, such as a vivid color or rapid stimulus onset. In a comprehensive review of recent research on how reward affects attention, Chelazzi et al. (2013) concluded with the sentence, “It may seem… paradoxical that learning principles…developed to explain overt behavior within a theoretical framework that was skeptical about the hidden and impalpable intricacies of cognition now appear to be perfectly suited to account for reward-based changes in attentional priority in the short and in the long term.”

Our procedure is a cognitive version of the “two-armed bandit” choice experiment (Estes, 1976; Shanks et al., 2002). In the choice version, the subject has two options on each trial. Each pays off at a fixed probability; for example one may provide a reward on 25% of the trials, while the other offers a reward on 75% of the trials. Under these conditions, choice probabilities often approximate the programmed reward probabilities (Estes, 1976; Fantino and Esfandiari, 2002). However, the optimal strategy is to choose the option that has the higher probability of reward on 100% of the trials. Thus, probability matching is anomalous from the perspective of rational choice theory (see Vulkan, 2000) but is consistent with a number of mathematical learning theories (e.g., Estes, 1976). The “discrepancy” suggests that psychological principles trump economic rationality. This is intriguing and has motivated 100s of two-armed bandit choice experiments. However, the results are mixed. On the one hand, many studies report probability matching, leading many researchers to conclude that this is the expected result (Gaissmaier and Schooler, 2008; Otto et al., 2011). On the other hand, there is evidence that probability matching is not a long-term stable equilibrium (e.g., Goodie and Fantino, 1999; Shanks et al., 2002). When the experiment includes feedback and/or incentives or proceeds for many 100s of trials, subjects tend to deviate from probability matching to maximizing (see Vulkan, 2000 and Shanks et al., 2002 for reviews). Consequently, we included an incentive and feedback conditions to test whether similar relations hold for attention allocation.

However, there is a more basic methodological issue. We used a single variable linear equation to model performance in the experimental procedure. The variable was set equal to the division of attention between the two simultaneously displayed visual stimuli. Thus, by solving the equation, we could calculate the allocation of attention. However, to properly interpret the results, performance in the procedure should approximate the assumptions of the model. The key assumption is that on each trial the subject attended to one of the two stimuli, but not both (or neither). For behavioral choice experiments, the analogy is that the devices for counting responses work properly. That is, the allocation equation in the present cognitive study can be considered analogous to the equipment used to tally responses in a behavioral study.

Just as we cannot say beforehand whether probability matching, maximizing, or some other pattern will emerge in the data, we cannot say in advance whether the calculations will prove valid. In dichotic listening tasks, the un-shadowed message makes some impression (Moray, 1959; Bentin et al., 1995) and, in their review of visual attention, Chun and Wolfe (2001) provide evidence that attention can be allocated simultaneously to multiple targets. Thus, attention may not be as constrained as is choice, and, accordingly, it may not be possible to arrange conditions so that our equation reliably measures the allocation of cognitive resources.

We should also point out that our measures and procedure allow for more than one cognitive bottleneck. One stimulus may have left a weaker “initial trace” or, in the process of attending to one stimulus, the “initial trace” of the other stimulus may have faded from memory. These are not mutually exclusive or exhaustive possibilities. We touch on “where” the limitations in cognitive processing in this experiment may have occurred in the Discussion Section of this paper. However, the primary goal of this research study is to quantitatively characterize the operating characteristics of cognition in a procedure that is analogous to a well-studied behavioral procedure.

## Experiment 1

### Materials and Methods

#### Participants

Forty-one undergraduate students (24 female, 17 male) from Boston College, ages 18–22, served as subjects. We used Boston College’s Sona Systems, a subject pool software program, to recruit subjects. Prior to the start of the experiment all participants signed a consent form according to the protocols established by the Boston College institutional review board for research. In addition subjects filled out a form that included questions regarding age, gender, year in school, major, GPA, SAT score, ACT score, home zip code, birth order, number of siblings, and several filler questions regarding food and music preferences. The subjects earned credit for Boston College Psychology Department research requirements for their participation. (This requirement could also be fulfilled by writing a short paper.) In addition, 21 subjects received feedback and money for correct responses, as described below. Four subjects did not complete Session 2. Thus, the data analyses are based on 37 subjects. The procedure and consent form were approved by the Boston College institutional review board.

#### Procedure

##### Stimuli

The experiment was conducted on a laptop computer. The screen measured 31.1 cm × 17.5 cm, and was set at a resolution of 1366 × 768 pixels. The subjects sat at their preferred distance from the computer, which we estimated at approximately 43–65 cm from the laptop screen.

**FIGURE 1:**
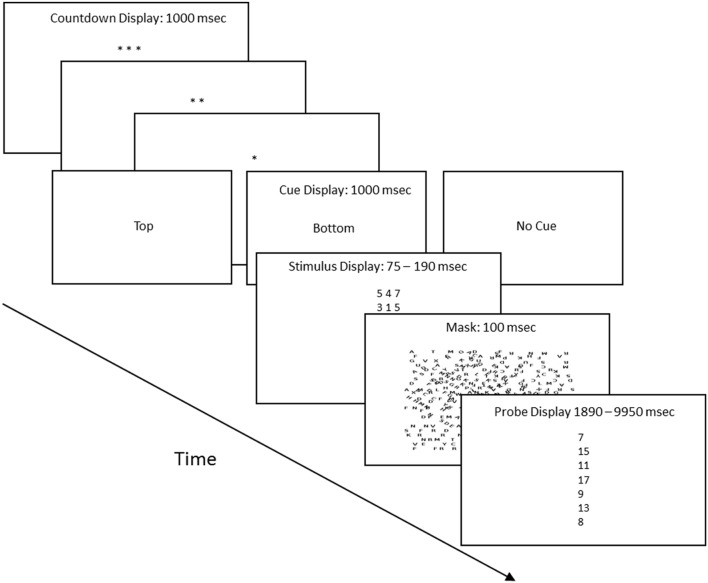
**Procedure flow chart (not to scale).** Each trial proceeded in four steps. (1) Preparatory count down, with each asterisk screen displayed for 1 s. (2) This was followed by the cue display, which indicated whether it was a cued or uncued trial, and if cued, whether the top row or bottom row of the stimulus held the three digits whose sum matched one of the sums in the probe screen. (3) The stimulus screen, whose duration was determined by the calibration procedure. The title also lists the 5th and 95th longest stimulus screen durations. (4) The probe screen, which was a vertical list of seven numbers. One matched either the sum of the three digits of the top row of the stimulus screen or matched the sum of the three digits of the bottom row of the stimulus screen. The title lists the 5th and 95th longest probe screen durations.

The session entailed 132 trials: 22 were cued and 120 were non-cued. Each trial proceeded in four steps: a preparatory count-down period, a cue display which indicated the type of trial, a stimulus screen, and a probe screen. **Figure 1** shows the corresponding displays. The countdown period began with three asterisks and ended with one, with an asterisk disappearing every second and a 100 ms inter-screen interval. Following the last asterisk, the screen displayed the words “top” or “bottom” for cued trials, or “no cue” for non-cued trials. “Top” and “bottom” identified the salient stimulus row for the 22 cued trials. Next, the stimulus screen displayed six digits arranged in two horizontal, parallel rows of three digits each. The digits were drawn from a list composed of the numbers 1–7 and were selected according to the following constraints: a row could not contain three instances of the same digit, the digits had to sum to a number on the interval 7–17, and each of the 11 possible sums had to appear equally often. The duration of the stimulus screen was calibrated individually for each subject as described below. **Figure 1** lists the 5th and 95th longest durations. The stimulus screen was followed by a 100 ms mask composed of upper case letters arranged in random orientations. Last, the probe screen listed seven numbers, one of which matched the sum of the three digits in the top row or the sum of the three digits in the bottom row of the preceding stimulus screen. The subject was asked to identify the matching sum. This could be done in two different ways. He or she could have attended to the row that provided the matching sum, or, given the multiple choice format, the subject could guess the correct sum. Assuming that subjects guessed when they failed to find the sum that they had just calculated (because they had attended to the row that did not contain the matching sum), response times for correct guesses should be longer than response times for correct responses mediated by attention. The subjects had up to 20 s to respond to the probe screen. **Figure 1** lists the 5th and 95th longest latencies for trials in which the subject correctly identified the matching sum.

In the stimulus screen each digit measured approximately 0.18 cm × 0.32 cm and each row of three digits measured 0.71 cm × 0.32 cm, with a space of 0.24 cm between each row. Thus, depending on the participant’s viewing distance, the entire stimulus array subtended visual angles of 0.95 × 1.17° to 0.63 × 0.78°, each digit subtended visual angles of about 0.27 × 0.43° to 0.18 × 0.28°, each row subtended angles of about 0.95 × 0.43° to 0.63 × 0.28°, and the border between the two rows subtended visual angles of about 0.95 × 0.32° to 0.63 × 0.21°. Visual angles of less than 5° are described as “small” or “narrow” (Goolkasian, 1991; De Cesarei and Codispoti, 2008). This judgment is based on the sizes of the fovea and foveola, which, according to standard texts, are approximately 5.0 and 1.2°, respectively (Wandell, 1995, n.d.). Thus, the entire stimulus array was well within the view of a fixed gaze.

##### Stimulus screen duration and calibration trials

At the start of each session, the experimenter conducted a series of calibration trials. The goal was to find the shortest stimulus duration that would reliably support accuracy scores of approximately 90% on cued trials, which is to say, in trials in which the subject knew beforehand which row held the three digits that added up to one of the sums listed in the probe screen. The calibration trials proceeded in blocks of 4, 8, or 16 trials; 75% were cued; and top and bottom cued trials were equally likely. All subjects were first tested at exposure durations of 600, 400, 300, 200, and 150 ms in descending order. These tests were four trials long. Next, the exposure durations were changed in smaller steps, and the number of trials was increased to 8 and/or 16 at each duration. In the final calibration phase, adjustments were made in yet smaller steps until the subject consistently had accuracy scores of at least 90% but less than 100% on top and bottom cued trials in 8 and/or 16 trial blocks.

##### Experimental session trials

The experimental session was divided into two parts with a rest period in between the two halves after Trial 66. Over the course of the session there were 22 cued trials and 110 uncued trials, in pseudo-random order. The stimulus exposure time was fixed at the value established by the calibration trials. The probabilities that the top row contained the matching sum were 0.1 (8 subjects but one did not complete Session 2), 0.25 (9 subjects), 0.5 (8 subjects but three did not complete Session 2), 0.75 (8 subjects), and 0.90 (8 subjects). The bottom row probabilities were the complements of these values. The order in which the top and bottom rows contained the matching sum was set by a list that was determined randomly with the constraint that the overall probabilities were as close as possible to the expected probability in each half session (55 uncued trials). On cued trials (11/half session), the probability that the top row contained a match was 0.55 in the first half of the session and 0.45 in the second half of the session.

##### Calculating the allocation of attention

Assuming that attention is a limited capacity and that the subject selectively attends to one but not both rows of the stimulus screen, a linear equation describes the relationship between obtained correct responses, attention allocation, and the probabilities that a stimulus is correct:

Expected⁢ % correct⁢ matches =PTp+PT(1−p)g+PB(1−p)+PBpg,

where *PT* is the probability that the top row is composed of the three digits whose sum matches one of the probe screen sums, *PB* is the probability that the bottom row has the matching digits, *p* is the probability that the participant attended the top row, *(1-p)* is the probability that the participant attended to the bottom row, and *g* is the frequency of correct guesses. For instance, if *PT* was set to = 0.75, and the subject attended to the top row on 75% of the trials, i.e., *p* = 0.75 (probability matching), and guessed correctly on trials that the unattended stimulus was the correct one as determined by chance (0.143), then the expected relative frequency of correct top row responses would be 0.589 or [0.75 × 0.75 + (0.75 × 0.25 × 0.143)]. Given these definitions we can solve Equation 1 for *g* and for *p.*

Let *PCT* equal the probability that there was a correct response when the top row was correct and analogously let *PCB* equal the probability of a correct response when the bottom row was correct:

PCT =((PTp)/PT)+((PT(1−p)g))/PT) =p+(1−p)g⁢

PCB =((PB(1−p))/PB)+(PBpg)/PB =(1−p)+pg.

Now add Equations 1a, 1b and solve for *g*:

g =(PCT+PCB)−1.

Next substitute Equation 1c for *g* in Equation 1a and solve for *p:*

p =(PCB−1)/(PCT+PCB−2).

Thus, on the basis of the programmed probabilities of top and bottom row matches and the numbers of correct responses, it is possible to calculate the relative amount of cognitive processing that each stimulus attracts and the probability of a correct guess on trials that the unattended to stimulus was correct.

##### Adjusting the model for errors

*Equations 1* to *1d* imply that the subjects never fail to attend to the stimuli and never make addition errors. In order to include the more plausible assumptions that there will be occasional lapses of attention and errors, we added a term for accuracy to the equations. We set accuracy on uncued trials equal to the obtained probability of a correct response on cued trials. Our thinking was that the best empirical estimate of errors or lapses in attention was performance on cued trials. On these trials the subject knows which stimulus to attend to so that an incorrect match reflects an error in adding or a lapse in attention. Accordingly, we multiplied the equations for correct top and bottom row responses on uncued trials by the probability of a correct response on cued trials (this was typically a number between 0.90 and 1.0). The resulting solutions for *g* and *p* have the same form as *Equations 1c* and *1d* except that *A* (accuracy) substitutes for 1.0 and *2A* substitutes for 2.0. For example, if accuracy were perfect (*A* = 1.0) then the two approaches yield the same solutions.

g =(PCT+PCB)−A

p =(PCB−A)/(PCT+PCB−2A)

When the accuracy rates on cued trials for the two rows differ, the equation for *p* is quadratic, as described in the Appendix, Part 1. However, this empirical approach to including errors in the model has a potential pitfall. Accuracy on cued and uncued trials may differ. When this happens it is possible to obtain specious values of *p* or *g* (i.e., values of *g* that were less than 0.0 or values of *p* that were less than 0.0 or more than 1.0). This occurred infrequently, and in almost all cases was resolved by using larger sample sizes (e.g., two sessions) to calculate guess rates and attention allocation (thereby reducing the difference between cued and uncued trial accuracies due to random variation). Part 3 of Appendix provides the details of the calculations along with the rule for using the quadratic model of the procedure (*Equations A1c, A1d*).

##### Feedback and payment for correct answers

At the end of the session subjects were told their overall percentage of correct responses. In addition, half of the participants were given an incentive for correct answers and within session feedback. Every five uncued trials, a screen displayed the number of correct responses in the last five uncued trials. At the end of the session, these same subjects also received 2–12 dollars, based on the number of correct responses. We provided both information (feedback) and an incentive to increase the salience of correct responses.

##### Statistical analyses

To analyze the possible differences between probability matching and maximizing, the allocation values were transformed to deviations from maximizing. Many of the values were close to 0.0 and deviated markedly from normality as determined by normal probability plots and the Shapiro–Wilk test (Stata). A square-root transformation normalized the values by these two criteria.

### Results

#### Attention Allocation

**FIGURE 2:**
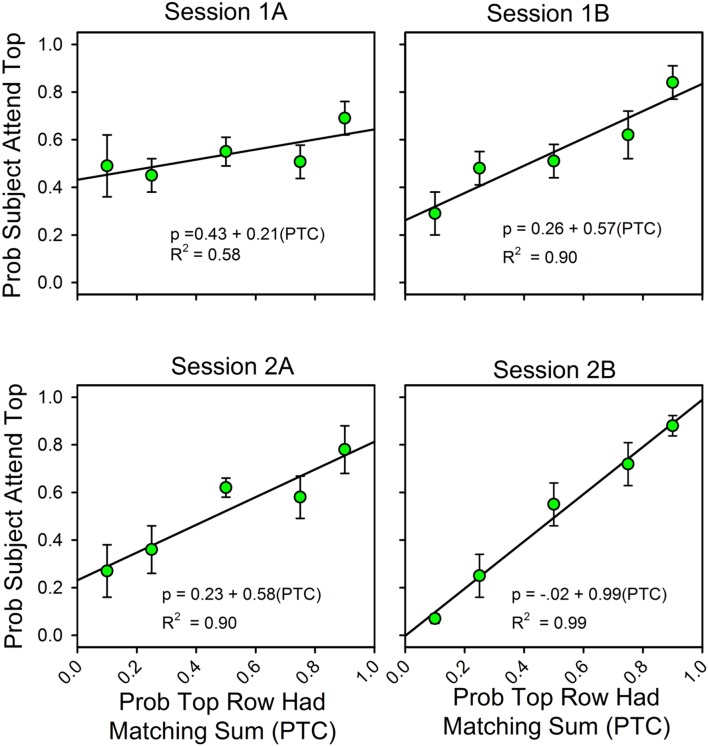
**The probability of finding the correct response predicts the allocation of attention.** Error bars show standard errors. (1A,B) refer to the first and second halves of Session 1; (2A,B) refer to the first and second halves of Session 2. Each panel includes the parameters for the best fitting line for the relationship between attention allocation and the probabilities that the top row and bottom row stimuli contained the digits for a correct response. As a function of experience, attention allocation shifted toward the stimulus that was more likely to predict the correct answer.

**Figure 2** summarizes changes in the allocation of attention over the course of the two sessions. On the *x*-axis is the likelihood that the sum of the three numbers in the top row was one of the seven answers listed in the probe screen. “A” and “B” refer to the first and second half of each of the two sessions (66 trials each). On the *y*-axis is the probability that subjects attended to the top row during the stimulus screen on uncued trials, as calculated by the equations. The Appendix provides the calculation details.

Over the course of the two sessions, attention shifted in favor of the row that was most likely to contain the matching sum. The slopes of the fitted lines increased from 0.21 to 0.99, and the intercepts decreased from 0.43 to 0.01, so that in the last half of the second session the equation for the best fitting line relating the allocation of attention to the likelihoods of finding the correct answer in the top and bottom rows was *y* = 0.01 + 0.99*x*. In support of the graphs, an ANOVA showed a statistically significant change in slopes as a function of session. Attention allocation values were the dependent variable, the predictiveness of the uncued stimuli was a between-subject factor, and session block was a within-subject factor. Stimulus predictiveness was a significant factor [*F*(4,32) = 9.53, *p* < 0.0005, $\eta_{p}^{2}$ = 0.544], and there was a significant stimulus predictiveness session block interaction [*F*(12,96) = 3.70, *p* = < 0.0005, $\eta_{p}^{2}$ = 0.316]. This corresponds to the increase in slope and decrease in intercept across half-session blocks. Further analysis revealed that the interaction had a significant linear component [*F*(4,32) = 5.57, *p* = 0.002, $\eta_{p}^{2}$ = 0.411] and a significant cubic component [*F*(4,32) = 2.77, *p* = 0.044, $\eta_{p}^{2}$ = 0.257], but not a significant quadratic component [*F*(4,32) = 0.413, *p* = 0.798, $\eta_{p}^{2}$ = 0.049]. Session itself was not significant [*F*(3,96) = 0.733, *p* = 0.535, $\eta_{p}^{2}$ = 0.022], which means that the average allocation values did not change over time (as expected).

##### Payment and feedback

**FIGURE 3:**
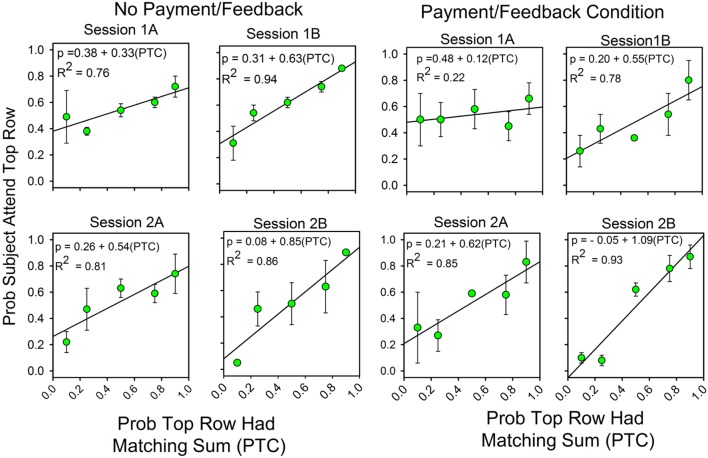
**Feedback enhances the correlation between attention allocation and the probability of finding the correct response.** The format of **Figure 2** is the same as in **Figure 1**. The four panels on the left show the results for the subjects without payment/feedback. The four panels of the rights show the allocation results for the subjects with payment/feedback. In the second session payment/feedback encouraged deviations from probability matching which increased the number of correct responses.

**Figure 2** combines the results for the subjects with and without feedback. **Figure 3** shows the results for these two groups separately. The graphs show differences in the second half of the second session when the predictiveness of the two stimuli were in ratios of either 3:1 or 1:3. For the no payment/feedback subjects, deviations from probability matching were biased in favor of 50:50 (“under-matching”). For the payment/feedback subjects, the bias was for maximizing. ANOVA showed that the differences were statistically significant. To increase the sample size, we pooled the 1:3 with the 3:1 results and likewise pooled the 1:9 with 9:1 results. This was done by recalculating the allocation probabilities as differences from the maximizing solutions, 0.0 when top row predictiveness was less than 0.50, and 1.0 when top row predictiveness was greater than 0.50. The 1:1 results were not included in this analysis, because in this condition the frequency of a correct responses is independent of the allocation of attention and thus not relevant to whether subjects maximized. The new dependent variable, then, is the difference from maximizing, and the predictors are presence or absence of payment/feedback and the predictiveness of the stimuli. There were 15 subjects in the no-feedback condition and 17 in the feedback condition.

Predictiveness (3:1/1:3 vs. 9:1/1:9) was a significant factor [*F*(1,28) = 6.560, *p* = 0.016, η^2^ = 0.190], payment/feedback was a significant factor [*F*(1,28) = 4.356, *p* = 0.046, η^2^ = 0.135] and the interaction between predictiveness and payment/feedback was significant: [*F*(1,28) = 6.135, *p* = 0.020, η^2^ = 0.180]. Further analyses showed that the pairwise comparison for the subjects at 3:1/1:3 probabilities was significant [*F*(1,28) = 11.15, *p* = 0.002, η^2^ = 0.284], but the pairwise comparison at the 9:1:1/9 was not significant [*F*(1,28) = 0.071, *p* = 0.791, η^2^ = 0.003]. These last two results correspond to the relatively larger differences between the two groups at top row probabilities of 0.25 and 0.75 and the relatively small differences at 0.10 and 0.90 (see the panels labeled “2b”).

#### Response Latencies at the Less Predictive Stimulus Were Longer

**FIGURE 4:**
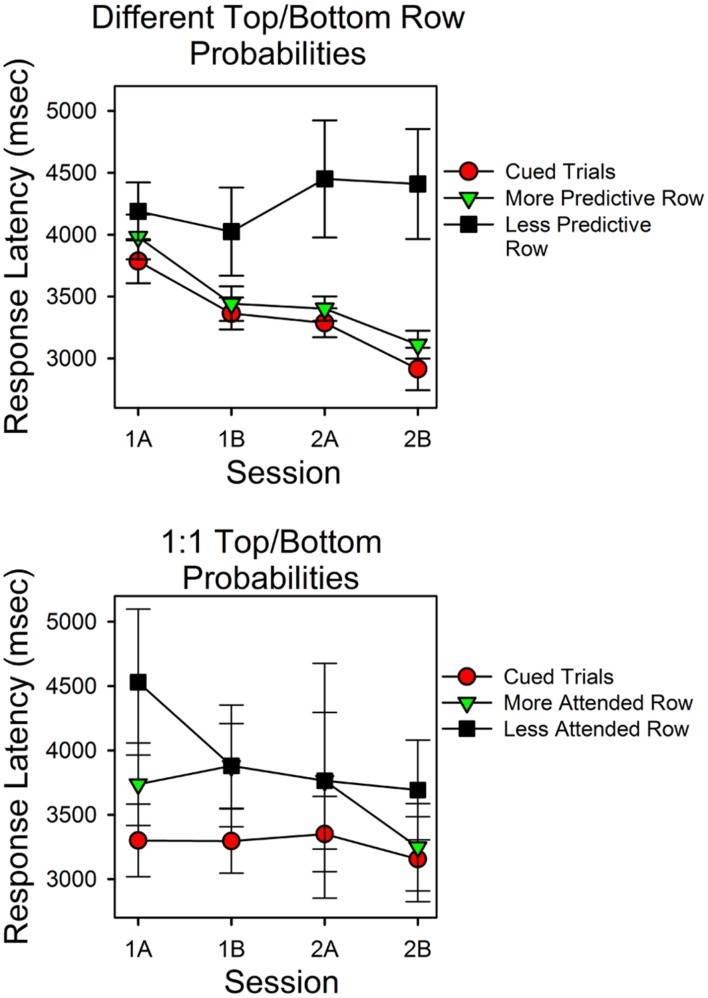
**Latencies for correct responses provide a test of the validity of the calculations.** The two panels show the average times for correct responses as a function of session block and whether a cue signaled the correct stimulus. In the upper panel the probabilities for top row and bottom row matches differed. In the lower panel the probabilities of a matching sum in the top and bottom rows were the same (0.50). The filled red circles show the average response times for correct responses on cued trials. The assumption that subjects could attend one row of the stimulus array but not both rows predicts the pattern of changes in response times and the longer response times for the less predictive row.

**Figure 4** shows response latencies for correct responses for cued trials and uncued trials. On the *x*-axis is session block; on the *y*-axis are latencies for the three types of trials. The top panel shows the results for the subjects for whom the predictiveness of the top and bottom stimuli differed; the bottom panel shows latencies for the subjects in the condition in which the predictiveness of each stimulus was 0.50.

The top panel shows that latencies differed as a function of whether the correct stimulus was cued, predictiveness and trial block. Latencies were shortest in cued trials (filled red circles) and longest in the uncued trials in which the less predictive stimulus was correct (filled black squares). Correct response latencies for the more predictive uncued stimulus overlapped with cued trial latencies, and both decreased as a function of session block. In support of these observations, a within subject ANOVA revealed a significant type of trial effect [*F*(2,42) = 5.614, *p* = 0.007, $\eta_{p}^{2}$ = 0.211] and a significant session block effect [*F*(3,63) = 5.844, *p* = 0.001, $\eta_{p}^{2}$ = 0.218]. Further analyses revealed that the session block effect had a significant linear component [*F*(1,21) = 11.72, *p* = 0.003, $\eta_{p}^{2}$ = 0.358], and that in the first and second half of the second session, latencies in cued trials and the more predictive uncued trials did not differ significantly [*t*(31) = -1.170, *p* = 0.251; *t*(31) = -0.955, *p* = 0.347], whereas latencies in cued trials and in the less predictive uncued trials differed significantly [*t*(28) = -2.55, *p* = 0.016; *t*(26) = -3.344, *p* = 0.003]. The session-block type-of-trial interaction was not significant [*F*(6,126) = 2.418, *p* = 0.213, $\eta_{p}^{2}$ = 0.063]. As outlined in the Discussion, the simplest hypothesis that explains the different trends in response latencies is that correct answers mediated by guesses were more likely in uncued trials in which the less predictive stimulus was correct.

The bottom panel shows the latencies for correct responses for the condition in which the top and bottom row probabilities were both 0.50. In this condition, we grouped uncued trial response latencies as a function of which stimulus had the most correct responses (since stimulus predictiveness did not differ). Response times at the cued stimulus were consistently shorter than those in uncued trials. In uncued trials, latencies associated with the stimulus that attracted more correct responses tended to be shorter, and in the second half of the second session, these times overlapped substantially with cued trial response times. However, the standard errors were relatively large in this condition. Consequently, type of trial was not a significant factor [*F*(2,8) = 4.061, *p* = 0.061]. Similarly, response times did not differ significantly as a function of session block [*F*(3,12) = 1.338, *p* = 0.308].

#### Did individual Differences in Stimulus Array Durations Affect Performance?

**Table 1** lists the average exposure times, average correct guess rates, average accuracy scores, and the correlations of the latter two measures with exposure times. Recall that at the start of each session, we calibrated the duration of the stimulus array to the shortest time that supported accurate performance on cued trials. Since the exposure times could differ in Sessions 1 and 2, the analysis is for each session separately.

**Table 1:** Experiment 1 stimulus exposure time, correct guess rate, accuracy.

	Exposure time (avg/SD, ms)	Correct guess rate	Accuracy cued trials
Session 1	139.9/55.4	0.162/0.11	0.898/0.09
Correlations with exposure time (*r/p)*		0.084/1.00	0.130/1.00
Session 2	130.6/35.8	0.162/0.10	0.908/0.10
Correlations with exposure time (*r/p*)		-0.151/1.00	-0.255/0.38


**Table 1** shows that the average calibrated exposure times, correct guess rates, and accuracy scores on cued trials were similar in the two sessions. The correlations between exposure time, accuracy, and correct guess rate were small and not statistically significant. This means that subjects with longer stimulus exposure times were not necessarily better at guessing the answer at the unattended to stimulus nor more accurate in adding up the three digits. Notice that the average calculated guess rates in Sessions 1 and 2 were not significantly different than the expected value of 0.143 according to paired *t-*tests in Sessions 1 and 2: *t*(37) = -1.106, *p* = 0.276; *t*(37) = - 1.117 *p* = 0.271.

#### Attention Allocation for Strict Adherence to the Simplest Assumptions (*A* = 1.0, *g* = 0.143)

**FIGURE 5:**
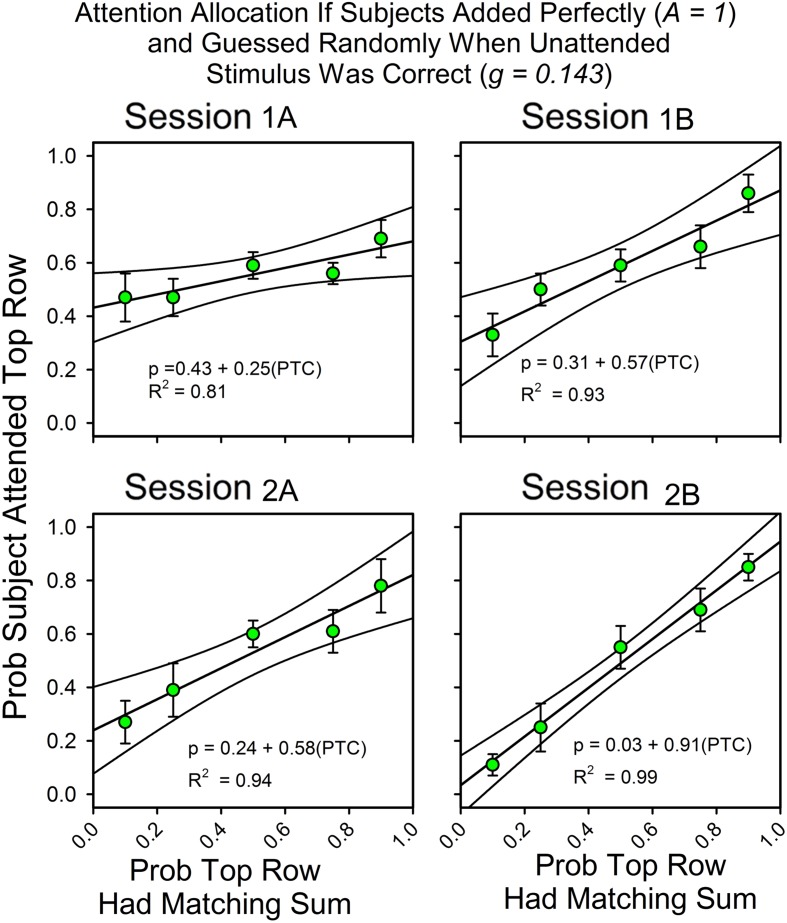
**Attention allocation for subjects who met perfectly the assumptions of the equations for calculating attention.** Attention allocation as a function of session and the probabilities of (top and bottom) row matches for subjects that added the three digits perfectly on every trial (*A* = 1.0) and whose correct guess rate at the unattended to row was exactly equal to the expected value (0.143).

In **Figures 2** and **3**, the obtained accuracy scores in cued trials were used to estimate attention allocation and correct guess rate in uncued trials (e.g., *Equation 2b*). However, our approach would be simpler if we calculated attention allocation according to the assumptions that the subjects always attended to the stimulus array, had no usable knowledge of the unattended to stimulus, and never made an arithmetic mistake. **Figure 5** shows the calculated allocation results for this simpler approach. The changes in slopes, intercepts and even the pattern of deviations from the fitted straight lines are about the same as when the observed accuracy rates in cued trials were used to calculate attention allocation.

### Discussion

If the quantitative principles that govern the allocation of choice also govern the allocation of attention, then in this experiment attention should shift systematically so as to approximate the probabilities of a correct response, as predicted by probability matching, and might also continue to shift yet more so as to increase the number of correct responses as predicted by maximizing. Moreover, if the determinants of the allocation of attention and the allocation of choice overlap, the tendency to maximize should be greater in the payment/feedback condition. However, before discussing these issues, there is the more elementary question of whether the procedure worked as intended. The calculations were based on the assumption that the subjects were able to attend to one stimulus (either the top or bottom row of digits) but not both. Do the data support these assumptions?

### Did the Procedure Work?

Correct guess rate, accuracy scores, response latencies, and calculation provide the evidence needed to determine if the assumptions that generated the model were met. If a subject attended to but one stimulus then the correct guess rate should be 0.143. For the same reason, there should not be a correlation between how long the stimulus screen was displayed and correct guess rate. If the subject had sufficient time to process one stimulus, then accuracy should be rather high, taking into account occasional lapses of attention and/or mathematical errors. If the subjects learned to attend to the stimulus that was more predictive then correct guesses must become relatively more frequent at the less predictive stimulus. Similarly, logic says that the latencies for these two different types of correct responses should differ. For example, imagine a subject that attended the more predictive stimulus exclusively. All correct responses at the more predictive stimulus would necessarily be mediated by attention, whereas all correct responses linked to the less predictive stimulus would have to be mediated by guessing. However, the subject would only know that they should guess if they had first reviewed all seven possible answers on the probe screen and discovered that the sum they had calculated was not the one that the computer had selected. They had attended to the wrong stimulus–and it is likely that guessing required some additional time as well. Thus, correct responses mediated by guessing should take longer, and the differences should increase as the subjects learn to attend to the more predictive stimulus. By the same sort of reasoning, latencies for correct responses at the more predictive stimulus should decrease and perhaps eventually approximate the cued response latencies. Finally, if the conditions that established the calculations were met then it should be possible to approximate the results by re-doing the calculations with accuracy set to 1.0 and correct guess rate set to 0.143. We have, then, several ways to check whether the procedure worked.

Inspection of the figures, statistical tests and the hypothetical calculations show that performance was consistent with the assumptions that were used to formulate the mathematical model of performance in this procedure and their behavioral implications. Correct guess rate did not differ significantly from the expected value of 0.143. Accuracy was about 0.90. Response times shifted in the predicted manner. The latencies for correct responses at the less predictive stimulus were longer, and they did not decrease as a function of session block. In contrast, the latencies for correct responses at the more predictive stimulus decreased as a function of session block and overlapped with the cued trial latencies. Response latencies were not well differentiated in the 1:1 condition, but here predictiveness was the same. **Figure 5** shows the results if accuracy had been perfect and correct guess rate had been exactly the expected value. The hypothetical results closely approximate the actual results. Each of these observations was supported by statistical tests. Session and stimulus conditions were significant predictors of changes in the allocation values and response latencies, and even feedback made a statistically significant difference, even though there was little room for maximizing deviations. The effect sizes were typically large, according to conventional standards. In sum, there were several ways of measuring whether the conditions for calculating attention were met; in each case the results passed the test.

The validity tests say it is reasonable to interpret the findings in terms of the question that motivated the study. Do the results support the idea that similar principles govern the allocation of overt behavior, as in choice experiments, and cognition, as in this selective attention study? Attention allocation approximated the probability matching predictions, as in behavioral studies, and deviations were predicted by maximizing. The correlates of maximizing in behavioral studies were the correlates of deviations toward maximizing in this study: feedback, monetary incentives, and continued exposure to the contingencies (e.g., Vulkan, 2000; Shanks et al., 2002).

We have not, though, identified the mediating allocation principles with any precision. For example both local and global maximizing strategies, such as the operant matching law (Herrnstein, 2000) and consumer choice theory (Baumol and Blinder, 1994), predict that subjects will deviate from probability matching toward exclusive preference in two-armed bandit choice procedures. Consequently, to obtain a more precise understanding of the principles that control the allocation of attention, we will have to modify the present procedure in ways that discriminate between these two competing theories.

### Variability and Sample Size

The equations for calculating attention allocation and correct guess rates can produce specious values [e.g., a correct guess rate (*g*) of less than 0.0 or a value of *p* greater than 1.0 or less than 0.0] if accuracy on cued and uncued trials differ and the subject attends one of the stimuli on most or all trials see Part 3 of the Appendix for details. Sample size made a difference. When the estimates of accuracy were based on session samples, 22 cued trials, the equations produced values of *p* that were less than 0.0 or greater than 1.0 in 21 of 148 cases. In contrast, when accuracy was based on the pooled (two-session) results, the number of specious results was cut in about half (10/148). This suggests that accuracy on cued and uncued trials was similar, and that specious results were due to random variation. In the remaining ten cases, we estimated the allocation of attention under the assumption that accuracy was perfect (*A* = 1.0) and the correct guess rate was the expected value (*g* = 0.143). **Figure 5** shows that these assumptions yield very similar estimates of attention allocation as estimates based on the obtained accuracies. Session data yielded four specious estimates of *g*, and pooled session data yielded no specious estimates of *g.* We used smaller samples sizes and empirical estimates of accuracy since they offered the most information about changes in attention over the course of the two sessions. However, data analysis and the presentation would have been simpler, albeit less informative, if we had restricted the analysis to whole sessions or to pooled Sessions 1 and 2 results.

### Conclusion

The results are consistent with the assumptions of the mathematical aspects of our approach for calculating the allocation of attention and with research on choice. The graphs and statistical analyses show strong and systematic correlations between the dependent measures, predictiveness and practice, and even feedback made a statistical difference even though there was not that much room for maximizing in the 9:1/1:9 conditions. Nevertheless, the mathematical model for calculating attention allocation is new so that it would be useful to provide more data regarding how well performance in our procedure supports its assumptions. Consequently, we ran a second study. The primary goal was to test the generality of the results summarized by **Figures 2**–**5**. In addition, we included three different feedback/incentive conditions. One provided a monetary incentive for each correct response; one provided the monetary incentive plus a record of the number of correct responses in the just previous ten responses; and one provided no incentive and no information on correct responses. The goal was to test whether explicit feedback and incentive had more of an impact on attention allocation than incentive alone, perhaps interacting synergistically.

## Experiment 2

### Introduction

Experiment 2 has two sets of goals. First, will it be possible to replicate the methodologically relevant results. The most important are those that follow from the assumptions of the equations. Namely that the correct guess rate approximates the expected value for a subject that has no useful knowledge of the unattended stimulus, that latencies for correct responses reflect whether guessing or attention mediated the response, and that accuracy on the cued trials remains at a high level over the course of the session. Second, Experiment 2 tests whether predictiveness and practice remain robust predictors of the shift in attention, and whether feedback and incentives predict deviations from probability matching toward maximizing.

The predictiveness of the top row was set at 0.20 or 0.80. For each probability, there were two payment/feedback conditions and one no payment/feedback condition, as described in the following Section “Materials and Methods”. On the basis of Experiment 1, we predicted that attention would gravitate toward the more predictive stimulus, that allocation values would differ as a function of payment/feedback, and that subjects would be more likely to deviate from probability matching toward maximizing in the payment/feedback conditions.

### Materials and Methods

#### Participants

Sixty-seven volunteers served as subjects. Forty were females, and 27 were males. The age range was 18–33, and the average age was 22 years. As this experiment was conducted in the summer, we used flyers posted on campus and summer school classroom announcements to recruit subjects. Since we were not able to provide subjects with course credit for participating in the study, we offered monetary compensation. In the payment/feedback conditions, each subject was offered $5.00 for participating plus money for correct responses as described below; in the no payment/feedback condition each subject was given 14.00/session for participating, which was about the average amount earned in the payment/feedback conditions. Note that in the no-feedback condition, payment was provided regardless of performance, just as in Experiment 1, subjects earned course credit regardless of their level of performance.

Prior to the start of the experiment all participants signed a consent form according to the protocols established by the Boston College institutional review board for research. One of the 67 subjects did not complete Session 2, and we omitted data for one subject who did not follow instructions. Thus, 65 subjects provided results in the statistical analyses. The procedure and consent form were approved by the Boston College institutional review board for research.

#### Procedure

The procedure was identical to that of Experiment 1, except for the payment/feedback conditions and how we recruited subjects. The incentive schedule was $0.10 for each correct answer on cued and uncued trials. Under this contingency random responding earned approximately $8.00 and the maximizing strategy earned approximately $14.00. In addition, one group of subjects also got feedback on their recent performance. Every ten trials, a screen displayed the number of correct cued and correct uncued responses in the just previous ten trials. In the no payment/feedback condition there was no monetary incentive for correct responses and no information regarding correct and incorrect responses.

### Data Analysis

**FIGURE 6:**
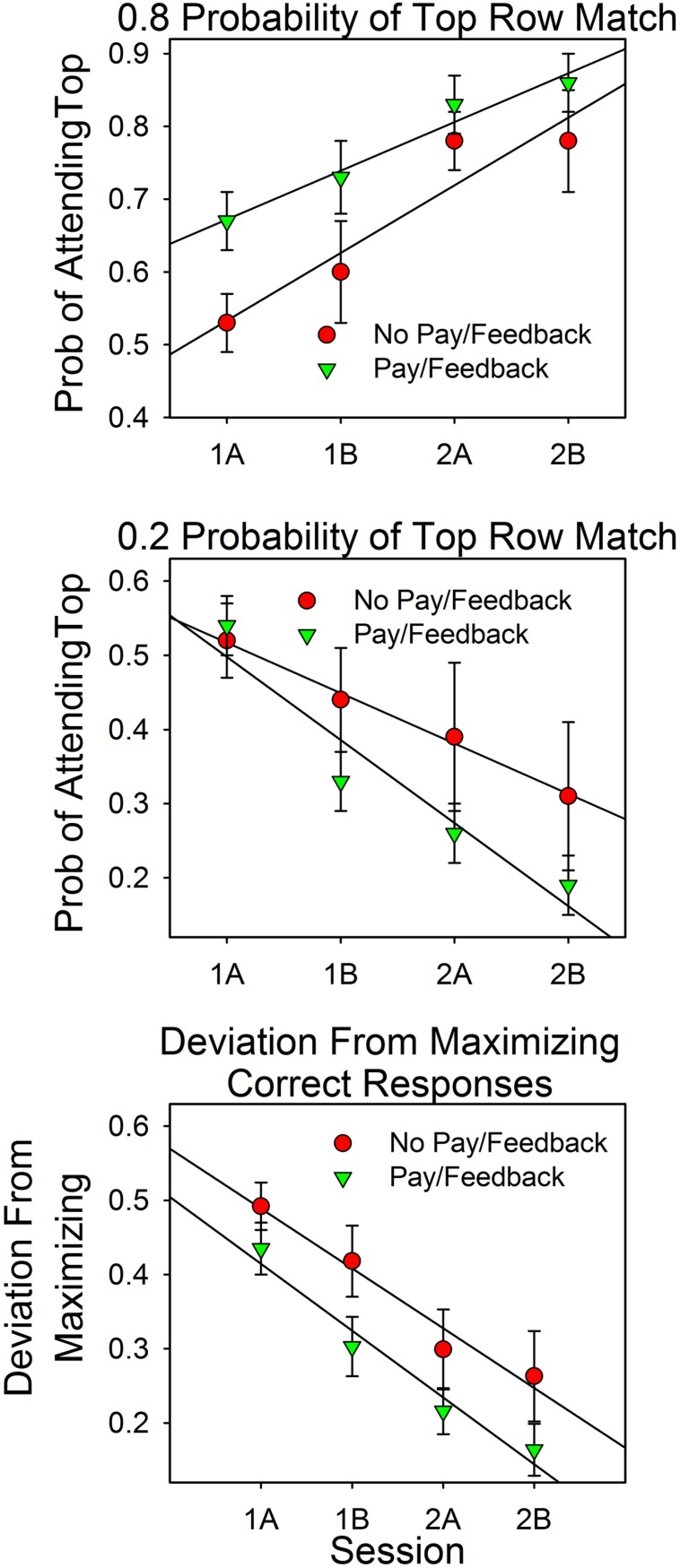
**Attention allocation and deviations from maximizing as a function of session and feedback.** The top panel shows the results for the 4:1 conditions. The middle panel shows the results for the 1:4 conditions. The bottom panel combines the results from the 4:1 and 1:4 conditions by reframing attention allocation as deviations from maximizing. For example, in the 4:1 condition, a top row attention allocation value of 0.80 is a 0.20 deviation from maximizing.

Data were analyzed as in Experiment 1. We combined the results from the two feedback conditions because they did not differ according to statistical tests. As in Experiment 1, deviations from maximizing (see the bottom panel of **Figure 6**) were often close to 0.0 and markedly non-normal, as determined by the Shapiro–Wilk test and normal probability plots (Stata). Also as in Experiment 1, a square root transformation of the differences reduced deviations from normality. Accordingly, statistical tests of deviations from maximizing were based on the square root transformed results.

### Results

#### Attention Allocation

**Figure 6** summarizes the results. On the *x*-axes are the first and second halves of Sessions 1 and 2. In the upper and middle panels, the *y*-axes are the relative amount of attention devoted to the top row stimulus on uncued trials in the 4:1 and 1:4 conditions, respectively. The lower panel shows the pooled results from the 4:1 and 1:4 conditions. On the *y*-axis is the magnitude of the discrepancies from the maximizing predictions. For example, a value of 0.20 implies an attention allocation value of 0.80 in the 4:1 condition and of 0.20 in the 1:4 condition.

The graph shows that attention allocation varied as a function of the predictiveness of the stimuli (either 0.8 or 0.2), practice (session block), and payment/feedback. The ANOVA results support these points.

As in Experiment 1, predictiveness and payment/feedback were between subject factors and trial block was a within subject factor. Predictiveness was statistically significant [*F*(1,61) = 53.66, *p* < 0.0005, $\eta_{p}^{2}$ = 0.468]. Feedback was evaluated in terms of the attention allocation values (the two top panels) and as deviations from maximizing. The attention allocation values canceled out since the analysis includes both 4:1 and 1:4 conditions, resulting in a very small *F* value: [*F*(1,61) = 0.010, *p* = 0.921]. In contrast, the interaction between predictiveness and feedback/payment, which distinguishes between the two predictiveness conditions, yielded a larger *F* value, but not large enough to pass the 0.05 criterion [*F*(1,61) = 3.554, *p* = 0.064, $\eta_{p}^{2}$ = 0.055]. However, when feedback was evaluated in terms of deviations from maximizing (bottom panel), differences between the payment/feedback subjects and the no payment/feedback subjects was significant at the 0.05 level: [*F*(1,63) = 4.05, *p* = 0.048, $\eta_{p}^{2}$ = 0.060].

Session block (trials) was a within-subject factor. Overall it was not significant since the 4:1 and 1:4 allocation values canceled out [*F*(3,183) = 1.09, *p* = 0.356, $\eta_{p}^{2}$ = 0.018]. However, the interaction with probability of a top row match, which distinguishes between the predictiveness of the stimuli, was significant: [*F*(3,183) = 24.48, *p* < 0.0005, $\eta_{p}^{2}$ = 0.318]. This correlation had a significant linear component [*F*(1,61) = 48.74, *p* < 0.0005, $\eta_{p}^{2}$ = 0.444]. Deviations from maximizing (bottom panel) decreased significantly as a function of session block [*F*(3,189) = 36.12, *p* < 0.0005, $\eta_{p}^{2}$ = 0.364], and this correlation also had a significant linear component [*F*(1,63) = 63.51, *p* < 0.0005, $\eta_{p}^{2}$ = 0.502]. The session block payment/feedback interaction was not significant [*F*(3,189) = 0.59, *p* = 0.620, $\eta_{p}^{2}$ = 0.009], as the parallel lines make clear.

### Response Latencies

**FIGURE 7:**
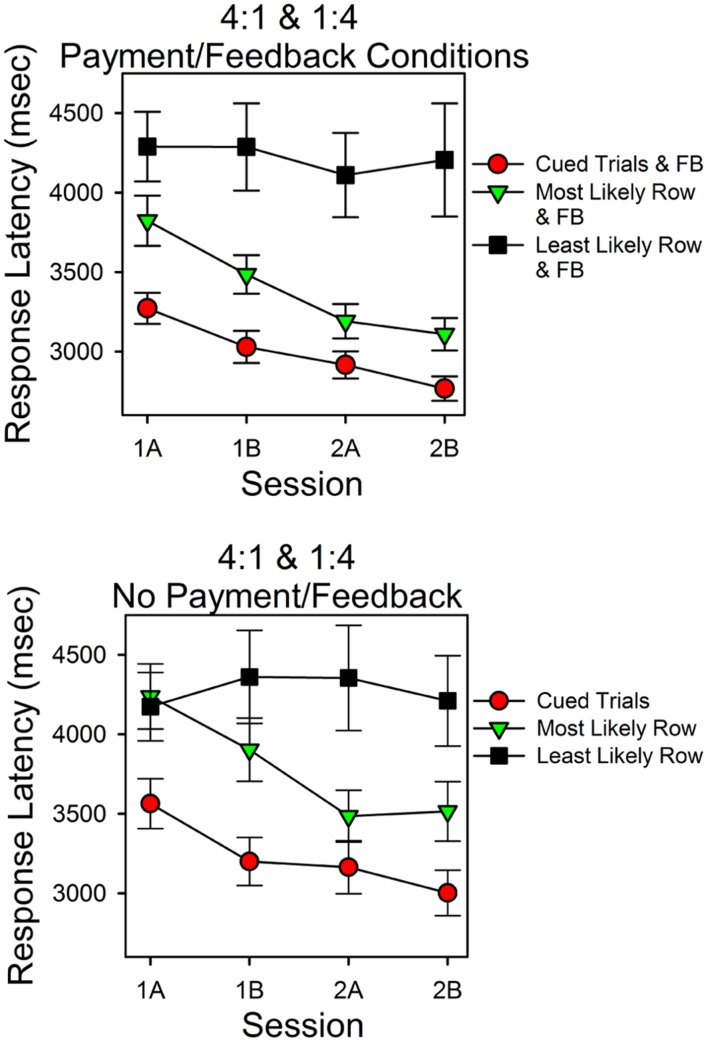
**Response latencies as a function of probability of finding the correct sum, cues, and feedback.** The format is the same as in **Figure 3**. As in Experiment 1 response latencies were a systematic function of the predictiveness of the stimuli and trials.

**Figure 7** shows the latencies for correct responses in Experiment 2 as a function of session block and type of trial. As in Experiment 1 there were three types of correct responses: those that occurred in cued trials (filled red circles), those that occurred in uncued trials when the more predictive stimulus was correct (filled green upside down triangles), and those that occurred in uncued trials when the less predictive stimulus was correct (filled black squares). The top panel is for the subjects in the payment/feedback conditions. The bottom panel is for the subjects who were not in the payment/feedback conditions. Consider the top panel first.

Response latencies varied as a function of session block and type of trial. The latencies for a correct response were shortest in the cued trials and longest in the uncued trials in which the less predictive stimulus was correct. Over the course of the two sessions, latencies for correct responses in the cued and uncued more predictive trials decreased. In contrast, latencies for correct responses in the less predictive uncued trials did not decrease. In support of these observations, ANOVA revealed that type of trial was a significant factor [*F*(2,70) = 33.05, *p* < 0.005, $\eta_{p}^{2}$ = 0.486] and session (practice) was a significant factor [*F*(3,105) = 2.73, *p* = 0.048, $\eta_{p}^{2}$ = 0.072]. Further analyses indicated a significant session (linear) type trial (quadratic) interaction [*F*(1,35) = 9.09, *p* = 0.005, $\eta_{p}^{2}$ = 0.206]. This corresponds to the different patterns of change across session block as a function of type of trial.

For the subjects who were not paid or given feedback, response latencies varied as type of trial and session block, as in the top panel, however, the differences were smaller. Within-subject ANOVA revealed that type of trial [*F*(2,40) = 17.93, *p* < 0.005, $\eta_{p}^{2}$ = 0.473] and session [*F*(3,60) = 2.935, *p* = 0.041, $\eta_{p}^{2}$ = 0.128] were significant factors. Further analyses indicated that the session effect included a significant linear component [*F*(1,20) = 7.46, *p* = 0.013, $\eta_{p}^{2}$ = 0.272], and, as in the top panel, there was a significant session (linear) type trial (quadratic) interaction [*F*(1,20) = 4.98, *p* = 0.037, $\eta_{p}^{2}$ = 0.200].

### Did individual Differences in Stimulus Array Durations Affect Performance?

We evaluated the correlations between the amount of time that the stimulus screen was displayed, the probability of a correct guess, and accuracy on cued trials in Sessions 1 and 2. **Table 2** summarizes the results.

**Table 2:** Experiment 2 stimulus exposure time, correct guess rate, accuracy.

	Exposure time (avg/SD, ms)	Correct guess rate	Accuracy cued trials
Session 1	131.6/29.05	0.132/0.10	0.947/0.05
Correlations with exposure time (*r/p)*		-0.290/0.063	-0.080/1.00
Session 2	117.08/32.01	0.139/0.09	0.939/0.12
Correlations with exposure time (*r/p*)		-0.220/0.268	0.032/1.00


As in Experiment 1, the exposure times, correct guess rates, and accuracy scores in cued trials were similar in the two sessions. The average exposure times differed by less than 15 ms (or about half a standard deviation), correct guess rates differed by about 0.01, and, similarly, accuracy on cued trials changed little. The correlations with exposure time were not large and were not statistically significant (see **Table 2** for values). According to paired *t-*tests, the average calculated guess rates in Sessions 1 and 2 did not differ significantly from the rate predicted by random guessing (0.143): *t*(63) = 0.917, *p* = 0.363; *t*(61) = 0.353, *p* = 0.726.

### Discussion

The results of Experiment 2 were consistent with the results of Experiment 1. Over the course of the two sessions, the probability of attending the top and bottom rows shifted according to the predictiveness of the stimuli. In the payment/feedback conditions, deviations from maximizing were significantly smaller than in the no payment/feedback condition. The average accuracy score on cued trials was 0.946, implying that subjects were able to successfully attend to one of the two stimuli. The correct guess closely approximated the theoretical value for a subject who had no usable knowledge of the unattended to stimulus (0.136 vs. 0.143). Latencies for correct responses were longer in trials in which the less predictive stimulus was correct, whereas in trials in which the more predictive stimulus was correct, response times decreased, approaching those when the subject was informed as to which stimulus was correct.

As in Experiment 1, the results indicate that subjects learned to attend to the more predictive stimulus, had little or no usable knowledge of the unattended stimulus, and guessed when they failed to attend to the correct stimulus. In other words, the results are consistent with the assumptions of the equations for calculating attention.

Also, as in Experiment 1, there were instances in which the equations gave an estimate of the allocation of attention that was less than 0.0 or greater than 1.0. This occurred in about 3% of the 260 calculations of *p.* See the Appendix for details.

## Experiments 1 and 2 Combined

**FIGURE 8:**
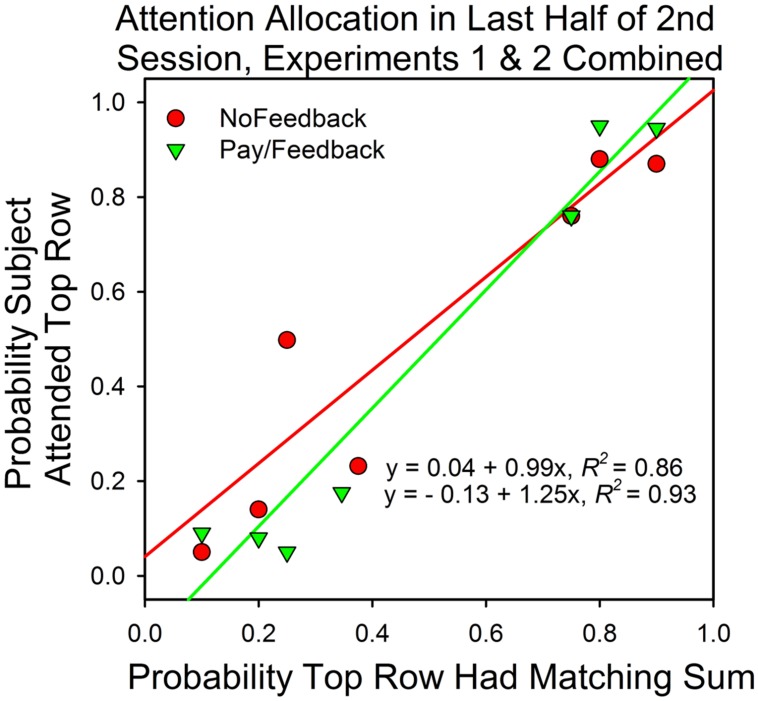
**Attention allocation in Experiments 1 and 2 as a function of predictiveness of the stimuli and payment/feedback.** The data points are the medians. Perfect probability matching implies a slope of 1.0 and an intercept of 0.0. Maximizing predicts a slope that is greater than 1.0.

**Figure 8** summarizes the results from Experiments 1 and 2. It shows the median attention allocation values in the payment/feedback and no payment/feedback conditions for the seven different probabilities of a top row match. In five of the six conditions in which the predictiveness of the top and bottom row stimuli differed, maximizing predicted deviations from probability matching when there was payment or payment∖feedback. For those subjects that received neither payment nor feedback, maximizing predicted deviations from probability matching in four conditions. Consequently, deviations from maximizing were smaller in the payment/feedback condition [*t*(95) = 2.64, *p* = 0.0.01].

## General Discussion

This study was motivated by three questions. Would it be possible to arrange conditions so that the equations for calculating the allocation of attention provided valid results? Would conditions that promote maximizing in behavioral choice experiments also promote maximizing in a cognitive procedure in which the “responses” were mathematically inferred? And would the results support the claim that there are general, quantitative laws in psychology that hold across sub-discipline borders?

### Validity

The results of both experiments are consistent with the assumptions that generated the equations. Accuracy scores averaged about 90% in Experiment 1 and about 95% in Experiment 2. Correct guess probabilities averaged about 0.16 in Experiment 1 and about 0.14 in Experiment 2–values which closely approximate the expected probability for a subject who guessed randomly on trials in which the attended-to row did not contain the matching sum. In both Experiments 1 and 2, response times for the less predictive stimulus were longer and changed little over trials, whereas response times at the more predictive stimulus decreased over trials, approaching the durations of response latencies on cued trials. This is precisely the expected pattern for a subject who is learning to attend to the stimulus with the higher likelihood of providing a matching sum and who has no usable knowledge of the contents of the unattended stimulus. The correlations between the duration of the stimulus screen, correct guess rate, and accuracy lead to the same conclusion. Correct guess rates approximated those predicted by random guessing, and accuracy was 90% or more (see **Tables 1** and **2**). As with latencies, this is precisely the expected pattern of performance for a subject who has learned to selectively attend to the more predictive stimulus at the cost of not attending to the less predictive stimulus.

### Conditions that Promote Maximizing in Choice Experiments Promoted Maximizing in the “Two-Armed Bandit” Cognitive Task

In two-armed bandit procedures, probability matching shifts toward maximizing as a function of feedback and practice (Goodie and Fantino, 1999; Shanks et al., 2002; West and Stanovich, 2003; Newell et al., 2013). This is the pattern we found if you substitute correct answers for reward and attention for choice. However, in this study the shift toward maximizing required substantially fewer trials than in choice experiments (e.g., Goodie and Fantino, 1999). Why this was so remains unexplored.

### Principles That Predict Value Driven Shifts in Attention

**Figure 8** shows that in five of the six conditions in which the top and bottom row probabilities of a correct answer differed, the median attention allocation values were closer to the predictions of maximizing (0.0 and 1.0) than of probability matching. To be sure, the differences were not large, but there was little room for large deviations and optimizing does not necessarily predict that the subjects should attend to just one option on all trials. In choice studies, subjects say that correct responses at the option that was less likely to payoff were more rewarding. In addition, sampling the less likely stimulus is reasonable in order to check if conditions have changed, and, reasonableness aside, there is evidence that variability is inherent to voluntary behavior (Neuringer, 2014). Thus, the optimal distribution of behavior in two-armed bandit procedures may be somewhat less than 100% in favor of the higher valued and/or more predictive stimulus and, perhaps, not that different from the allocation values observed in this study.

We have yet to investigate the processes that may have mediated the shifts toward maximizing. The choice literature offers several candidates: (1) global maximizing as assumed in economics (Baumol and Blinder, 1994); (2) feedback driven local maximizing processes, such as Herrnstein’s operant matching law and melioration principle (Herrnstein, 2000); (3) stochastic learning rules (Estes, 1976); and (4) simple, mechanical “rules of thumb” that automatically produce optimal or near optimal outcomes in some settings but not all (Heyman, 1982). Further research is needed to identify which, if any, of these principles guided attention in the present experiment (also see Shahan and Podlesnik, 2006, for a discussion of attention allocation and Herrnstein’s operant matching law).

### Limitations and Questions

To what extent was the unattended-to stimulus processed? For example, was the unattended to row of digits encoded but then forgotten as the subjects added the digits in the attended to row, as in studies in which later “stages” of cognitive processing become increasingly selective (Duncan, 1980 and Lavie et al., 2004)? Or was attention allocated so that the unattended digits remained relatively “unregistered” as in studies in which “early selection” is observed (Treisman, 1969; Lavie et al., 2004)? Possibly, a priming study would reveal greater knowledge of the unattended-to digits than the current procedure did.

It is not clear which aspect of the feedback conditions was most instrumental in promoting maximizing. Our goal was to test whether conditions that influence the allocation of choice also influence the allocation of attention. Accordingly we provided both information and incentives simultaneously in Experiment 1, as this is how the choice experiments work. In Experiment 2 there was an incentive condition without feedback and with feedback, however, these two conditions produced statistically indistinguishable results. What is missing is a feedback alone (no monetary incentive) condition. Consequently, the precise role of feedback and incentives in this procedure remain unclear. Likely both matter, and the results from Experiment 1 suggest that the importance of feedback and/or incentives will vary inversely with the absolute size of the differences in predictiveness for the two stimuli. For example, in the 9:1/ 1:9 conditions payment/feedback made no difference, whereas in the 3:1/1:3 conditions payment/feedback significantly increased deviations from probability matching toward maximizing.

## Conclusion

The methodological significance of this study is that it introduces a method for inferring the allocation of attention–a covert process–on a scale of 0.0–1.0. Although there are many methods for describing attention, none, as far as we know, calculate attention as the solution to an equation, thereby providing a numerical description that can vary continuously. This approach invites studies in which researchers can calculate the correlations between individual differences in the voluntary control of attention and individual differences in other psychological functions, such as higher order cognitive skills or obsessive tendencies. For instance, might subjects who more slowly learn to attend to the more predictive stimulus also tend to dwell on disturbing ideas? The theoretical significance of the results is that they are consistent with the idea that the same general principles govern attention and choice. Put somewhat differently, the results suggest that subjective value drives attention in much the same way that it drives choice. However, as noted in “*Limitations and Questions*”, the nature of the processes that mediated the relationship between reward and attention allocation in the present study remain an open question. Given the orderliness of the results in this study, this question should prove answerable.

## Author Contributions

GH developed the mathematical model, helped develop the procedure, helped analyze the data, helped write the paper. KG helped run the experiment, helped analyze the data, and helped write the paper. VL helped develop the procedure and test subjects.

## Conflict of Interest Statement

The authors declare that the research was conducted in the absence of any commercial or financial relationships that could be construed as a potential conflict of interest.

## APPENDIX

### Part 1: Calculating the Allocation of Attention When Accuracy for Top and Bottom Row Stimuli Differed

Accuracy rates for top and bottom rows sometimes differed on cued trials. To take this into account, we entered top and bottom row cued trial accuracy percentages (*A*1 and *A*2) separately in Eq. 2. This yields a quadratic equation in *p*:

PCT =A1p+(1−p)g

PCB =A2(1−p)+pg

g =PCT+PCB−A1p−A2(1−p)

Now substitute for *g* in Eq. *A*1b and set to 0.0

$$(A2-A1)p^{2}+(PCT+PCB-2A2)p+(A2-PCB)=0$$

We used the quadratic formula to solve the Eq. *A*1d, and then inserted the solution into the Eq. *A*1c in order to caculate the correct guess rate (*g*). Note that Eq. *A*1d reduces to Eq. 2b when top and bottom row accuracies are the same (as it should).

### Part 2: Calculating Attention Allocation When the Assumptions of the Model (Eq. 1) were Met Perfectly (*A* = 1.0 and *g* = 0.143)

If we substitute 1.0 for *A* and 0.143 for *g*, we can rewrite Eqs. 1a and 1b as:

p=(PCT−0.143)/0.857

p =(1−PCB)/0.857

Now add Eqs *A*2a and *A*2b:

2p =(PCT−PCB+0.857)/0.857

p =(PCT−PCB+0.857)/1.714

Although this equation assumes perfect accuracy, Figure 5 shows that its predictions differed little from those based on the empirically determinded accuracies (e.g., Eqs. 2a and 2b of the text and Eqs. A1c and A1d of this Appendix).

### Part 3: Procedures for Solving the Allocation of Attention Equations

We calculated *p* for half-session blocks (55 uncued trials each) for each subject in Experiments 1 and 2, using the session cued trial accuracy scores (22 trials) and the linear (Eq. 2) and quadratic models (Eq. *A*1). The two equations give identical results when top and bottom row accuracy were the same, and somewhat different results when top and bottom row accuracies differ. Inspection of the results suggested that we should use the quadratic form when the difference in top and bottom row accuracy was greater than 0.099. For instance, the linear and quadratic estimates of attention allocation differed by an average of 0.007 when the differences in top and bottom row accuracy were less than the 0.099 criterion, but differed by an average of 0.088 when the difference in top and bottom row accuracy was greater than 0.099. For about 75% of the cases, we used the linear model. The numbers are as follows.

There were a total of 408 half-session estimates of attention allocation (Experiment 1 = 148, Experiment 2 = 260). In 307 cases, we used the linear model (Eq. 2b). In 67 cases, we used the quadratic model (Eq. *A*1d). In 34 cases, neither equation gave an estimate of *p* on the interval 0.0 to 1.0. Inspection of the equations reveals that this can occur when cued trial and uncued trial accuracies differ and the subject attends largely or exclusively to one row. We dealt with this issue in a step-by-step manner according to the rule that empirical estimates of accuracy were preferred to an assumed level of accuracy. First, we redid the calculations using pooled Sessions 1 and 2 cued trial results. This doubled the sample size from 22 to 44 cued trials. This reduced the number of specious cases from 34 to 16. For the remaining 18 cases, we assumed that *A* = 1 and *g* = 0.143. Although this was not our preferred approach, Figure 5 shows that it produced results that were very similar to those based on empirical estimates of accuracy.

We calculated correct guess rate (*g*) on the basis of single session and pooled session results. As noted above, the average value of *g* was about the same in both sessions. Of the 204 single session estimates of *g*, 13 were less than 0.0, and for the 102 pooled session estimates, 6 were less than 0.0.
